# The methylome of *Biomphalaria glabrata* and other mollusks: enduring modification of epigenetic landscape and phenotypic traits by a new DNA methylation inhibitor

**DOI:** 10.1186/s13072-021-00422-7

**Published:** 2021-10-26

**Authors:** Nelia Luviano, Marie Lopez, Fleur Gawehns, Cristian Chaparro, Paola B. Arimondo, Slavica Ivanovic, Patrice David, Koen Verhoeven, Céline Cosseau, Christoph Grunau

**Affiliations:** 1 IHPE, Univ Perpignan Via Domitia, CNRC, Ifremer, Univ Montpellier, Perpignan, France; 2 Institut des Biomolécules Max Mousseron (IBMM), UMR 5247, CNRS, Univ Montpellier, ENSCM, Montpellier, France; 3 Epigenetic Targeting of Cancer (ETaC), CNRS FRE3600, Centre de Recherche et Développement Pierre Fabre, Toulouse, France; 4 Bioinformatics Unit, Netherlands Institute of Ecology (NIOO-KNAW), Wageningen, The Netherlands; 5 Epigenetic Chemical Biology (EpiChBio), Department Structural Biology and Chemistry, UMR 3523, CNRS, Institute Pasteur, 75015 Paris, France; 6 Department of Terrestrial Ecology, Netherlands Institute of Ecology (NIOO-KNAW), Wageningen, The Netherlands; 7 Centre d’Ecologie Fonctionnelle et Evolutive (CEFE), Univ. Montpellier, CNRS - Université Paul Valéry Montpellier - EPHE, 1919 Route de Mende, 34293 Montpellier Cedex 5, France

**Keywords:** DNMT inhibitors, 5-Methylcytosine, Mollusks, Invertebrates, Epigenetic inheritance, Epimutation

## Abstract

**Background:**

5-Methylcytosine (5mC) is an important epigenetic mark in eukaryotes. Little information about its role exists for invertebrates. To investigate the contribution of 5mC to phenotypic variation in invertebrates, alteration of methylation patterns needs to be produced. Here, we apply new non-nucleoside DNA methyltransferase inhibitors (DNMTi) to introduce aleatory changes into the methylome of mollusk species.

**Results:**

Flavanone inhibitor Flv1 was efficient in reducing 5mC in the freshwater snails *Biomphalaria glabrata* and *Physa acuta,* and to a lesser degree, probably due to lower stability in sea water, in the oyster *Crassostrea gigas*. Flv1 has no toxic effects and significantly decreased the 5mC level in the treated *B. glabrata* and in its offspring. Drug treatment triggers significant variation in the shell height in both generations. A reduced representation bisulfite-sequencing method called epiGBS corroborates hypomethylation effect of Flv1 in both *B. glabrata* generations and identifies seven Differential Methylated Regions (DMR) out of 32 found both in Flv1-exposed snails and its progeny, from which 5 were hypomethylated, demonstrating a multigenerational effect. By targeted bisulfite sequencing, we confirmed hypomethylation in a *locus* and show that it is associated with reduced gene expression.

**Conclusions:**

Flv1 is a new and efficient DNMTi that can be used to induce transient and heritable modifications of the epigenetic landscape and phenotypic traits in mollusks, a phylum of the invertebrates in which epigenetics is understudied.

**Supplementary Information:**

The online version contains supplementary material available at 10.1186/s13072-021-00422-7.

## Background

DNA methylation is an epigenetic mark that can be associated with changes in gene function without changes in the underlying DNA sequence [29]. Modifications in DNA methylation can be induced by the environment and some changes can be mitotically and/or meiotically heritable and/or are reversible [22, 63]. Some of these modifications can influence gene function by providing differential access to the underlying genetic information in cells, and thus may alter their phenotypes. Epigenetic marks, such as DNA methylation, may provide an additional dimension to inheritance, linked to, but different from genetic inheritance. Epimutations can be provoked directly by environmental stresses and contribute to rapid evolutionary changes, but unlike genetic variation, epimutations have higher rates and are reversible [9, 17]. Biochemically, DNA methylation is the modification of a DNA base, and is present in a diverse range of eukaryotic organisms, ranging from *fungi* to mammals [15]. One type of DNA methylation is cytosine methylation that is catalyzed by the DNA methyltransferases (DNMTs), enzymes that transfer the methyl group (–CH_3_) from the co-substrate *S*-adenosyl-l-methionine (AdoMet) to carbon-5 of cytidines, to form 5-methylcytidine (5mC) [61]. In vertebrates, DNA methylation occurs mostly on cytosines in a CpG context except for the CpG islands [84], whereas DNA methylation can also occur in CHH and CHG (H = A, T, C) context in plants [51]. Less is known about the methylation in invertebrates, though many species present DNA methylation in a CpG context [34].

DNA methylation is assumed to be evolutionary ancient, but its functions and patterns are very diversified. This is consistent with the notion of a dynamically evolving mechanism that can adapt to perform various functions [89] but having a common origin and being always part of an inheritance system [3]. Major differences in DNA methylation are observed among phyla [48]. In the animal kingdom, vertebrates have one of the highest levels of DNA methylation that is uniformly spread all over the genome and found in all sorts of genomic contexts such as gene bodies, gene promoters, intergenic regions, and repetitive DNA such as transposons [75] (“global methylation”). Only promoter sequences are generally unmethylated and methylation here has been demonstrated to modulate gene expression in *cis*. Methylation also affects DNA repair stability, splicing, imprinting, development, germ cell pluripotency and cell fate [72]. In contrast, in many invertebrates, a common type of DNA methylation is the “mosaic” pattern consisting in large domains of methylated DNA separated by large domains of unmethylated DNA [40]. Another pattern is a very low level [36] or a total absence of DNA methylation [3, 11]. When methylation is of mosaic type, 5mC is often found in genes (in exons and sometimes to a lesser degree in introns), a type of methylation also called Gene Body Methylation (GBM). GBM is considered as the ancestral form of DNA methylation [25]. Higher GBM is believed to be associated with active transcription in vertebrates and invertebrates, while promoter methylation in vertebrates is associated with repression of gene expression [71].

An important aspect of epigenetic marks is their inheritance. There is evidence in model species, mainly plants [44], that heritable variation in ecologically important traits can be generated through changes in DNA methylation and that these changes may be transmitted to future generations. Nevertheless, in contrast to plants and vertebrates, there is little evidence of transgenerational stability of DNA methylation in other clades such as Lophotrochozoa, that includes annelids, mollusks, bryozoans, brachiopods and platyhelminthes. Consequently, more evidence is needed about whether environmental-based DNA methylation changes can be inherited across generations in Lophotrochozoa and in here, we focused on this question in mollusks. DNA methylation has been relatively little investigated in mollusks as discussed in [24], where information is essentially based on data from two species: the pacific oyster *Crassostrea gigas* and the freshwater snail *Biomphalaria glabrata.* In the above-mentioned work, the authors distinguish the terms multigenerational and transgenerational. Multigenerational effect results from a direct exposure of the germline, gametes, or embryos to the environmental stress, while a transgenerational effect involves a germ line transmission between generations without direct exposure of the germ cells to the environmental stress [24]. In this work, we investigated these two mollusks’ species and added the previously unstudied *Physa acuta*, i.e., three molluscan models of medical, economic, and ecological importance.

The snail *B. glabrata* is the intermediate host of *Schistosoma mansoni*, the causative agent of schistosomiasis, a parasitic disease affecting 200 million people in 78 countries [58]. The interaction of these species is characterized by a phenomenon called compatibility polymorphism, meaning that some snail phenotypes can be infected by a specific parasite phenotype while others cannot [78]. It has been demonstrated that epigenetic alterations are involved in the *B. glabrata* parasite compatibility phenotype [49], even though contrasting results have been obtained by others [4, 74]. It remains, therefore, an open question whether epigenetic mechanisms play a role in the capacity of *B. glabrata* to produce phenotypic plasticity or variability. DNA methylation machinery components in *B. glabrata* include a maintenance DNMT (*Bg*DNMT1), a DNA/tRNA methyltransferase (*Bg*DNMT2) and a methyl-CpG-binding domain protein (*Bg*MBD2/3), *Bg*DNMT1 and *Bg*DNMT2 being probably responsible for the 5mC modifications [27, 32].

*Crassostrea gigas* is a mollusk of commercial importance and its phylogenetic position and life traits make this bivalve an ideal model to study the physiological, ecological, and evolutionary implications of DNA methylation [68]. In silico analysis revealed that genes predicted to be hypermethylated are generally involved in DNA and RNA metabolism and genes predicted to be sparsely methylated are involved in cell adhesion [69]. Similar results were found in *B. glabrata*: genes predicted to be methylated are associated with housekeeping functions and genes predicted to be poorly methylated are associated with inducible functions [27]. These findings suggest that DNA methylation has regulatory functions in genes implicated in stress and environmental responses meaning it could contribute to increase phenotypic plasticity in mollusks and/or produce potentially heritable phenotypic variation [69].

*Physa acuta* is one of the most widespread freshwater snail invaders [83] and is an occasional host of several human trematode diseases, including echinostomiasis and fascioliasis [21, 47]. Besides, it has been demonstrated to be a bioindicator species for its sensitivity to environmental contaminants [7, 62]. *P. acuta* has a short generation time that makes it a good model for multigenerational studies [73]. Studies about the impact of toxic compounds in the global DNA methylation of *P. acuta* and in its phenotypic traits [7] suggest that DNA methylation can play a role in the phenotypic plasticity of this snail; however, further work is needed to explore this hypothesis.

We borrowed an approach from cancer biology in which the use of DNMT inhibitors (DNMTi) has brought considerable advancements in the understanding of DNA methylation mechanisms but also in therapeutic approaches [35, 54, 64]. The most used DNMTi in many species is 5-azacytidine (5-AzaC) [5, 31, 57], nevertheless important advancements in the design of DNMTi have been done in the last years, notably in decreasing the toxicity and improving the specificity of these compounds [37, 64]. Further, 5-AzaC induces unstable and major side effects, e.g., it caused malformations and apoptosis in the fetal nervous system when administered into pregnant mice [81]. Therefore, we used the commercially available non-covalent nucleoside inhibitor, zebularine [14] and novel generation of non-nucleoside DNMT inhibitors that do not incorporate into DNA, and therefore, induce minimal side effects [23]. In addition, we evaluate if DNMTi-induced DNA methylation modifications are transmitted to their offspring. For global DNA methylation screening, we developed a simple, low cost, antibody-based method to measure DNA methylation levels over large sample numbers and requiring only small amounts of DNA. Our dot blot method and a commercial ELISA-based kit showed equivalent results. For genome-wide methylation profiling, we used epi-genotyping-by-sequencing method (epiGBS) [30, 82] a reduced representation bisulfite-sequencing technique that captures a percentage of the epigenome (1–5%) and we compared the results with a previous methylation information obtained by Whole-Genome Bisulfite Sequencing (WGBS) [1].

We tested two types of DNMTi with different mechanisms of action (Additional file 1: Figure S1). We used zebularine, a nucleoside analogue of cytidine that has proven to be an inhibitor of DNA methylation in human cancer cells [16] but differently from 5-AzaC, it does not form an irreversible covalent complex with the DNMTs [14] and two custom-made compounds (nitroflavanones) that showed in vitro inhibition activity against DNMT1 and DNMT3A-c in human cancer cell lines [65].

In summary, our results showed that flavanone Flv1 decreased 5mC level in the exposed generation and mostly in its progeny, it triggered variation in the morphometric traits in both generations and it did not show toxic effects. EpiGBS sequencing confirmed the genome-wide effect caused by Flv1 and allowed us to find Differential Methylated Regions (DMRs) between treatment and control samples. Furthermore, a parental effect was demonstrated by the presence of seven Differential Methylated Regions in Flv1-exposed snails and its offspring. Flv1-induced hypomethylation in the BGLTMP010125 *locus* was associated with reduced gene expression. Since Flv1 inhibitor demonstrated efficiency as DNMTi in *B. glabrata*, it was also tested in the two other mollusk species: the oyster *C. gigas* and the freshwater snail *P. acuta*, where it triggered also significant decrease of 5mC, suggesting that Flv1 can be used to modify methylation in other mollusk species.

## Results

### DNMTi Flv1 and Flv2 are suitable for pulse treatment in freshwater

**Fig. 1:**
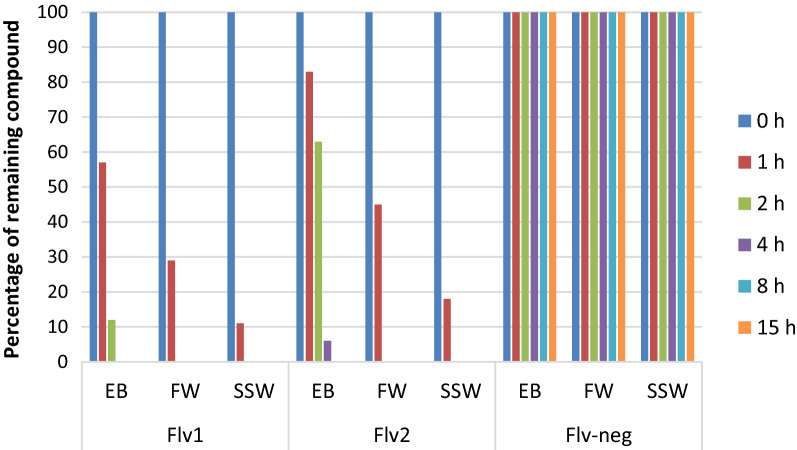
Flavanone chemical stability in three different media, enzymatic buffer (EB), freshwater (FW) and sea salt water (SSW). Stability was measured by HPLC at 100 µM of each compound in the three media

To induce changes in DNA methylation that were sufficiently strong to lead to phenotypic changes but not strong enough to have toxic effects, we intended to apply a brief DNMTi pulse of only a couple of hours. We, therefore, checked the stability of our new DNMTi in the water environment. The two active flavanones (Flv1 and Flv2) are moderately stable in the enzymatic buffer at pH 7.2 (EB), there was 12% of remaining compound after 2 h for the Flv1 and 6% of remaining compound after 4 h for the Flv2 (Fig. 1). Their stability decreased in freshwater at pH 7.8 (FW), with 29% of remaining compound after 1 h for Flv1 and 45% of remaining compound after 1 h for Flv2 (Fig. 1).

The lowest stabilities were found in sea salt water at pH 8 (SSW), 11% of remaining compound after 1 h for Flv1 and 18% of remaining compound after 1 h for Flv2. Interestingly, the negative flavanone compound (Flv-neg) was highly stable in the three conditions (EB, FW and SSW) (Fig. 1). Simply the addition of Flv1 and Flv2 to water environment can thus generate a precise pulse of inhibition of DNA methylation activity of 1–2 h without the need for replacing the water, avoiding confounding stress to the experimental setup.

### Flv1 blocks methyltransferase activity on *Bge* nuclear extracts

**Fig. 2.**
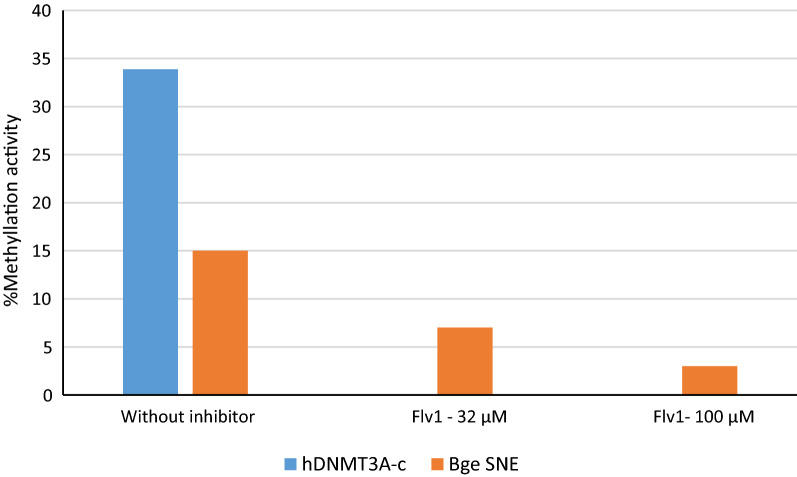
The level of methylation activity in *Bge* nuclear protein extract was 15%. After treatment with 32 µM and 100 µM of Flv1, methylation activity of *Bge* nuclear protein extract decreased to 7% and to 3%. Human hDNMT3a-c enzyme was used as a control (blue bar), and its methylation activity was completely inhibited by Flv1 treatment

Previous work had shown that Flv1 inhibits directly the DNMT activity in vertebrate cells [65]. We wished to establish such direct action on the methyltransferases in *B. glabrata*. To do so, we extracted soluble nuclear proteins from *Bge* cells and performed an in vitro enzyme inhibition assay. We showed that methylation activity of the *Bge* nuclear protein extract was inhibited by 55% and 78% after treatment with 32 µM and 100 µM of Flv1, respectively (Fig. 2).

### Dot blot densitometry allows for cost-efficient screening of 5mC global level in large sample numbers

**Fig. 3:**
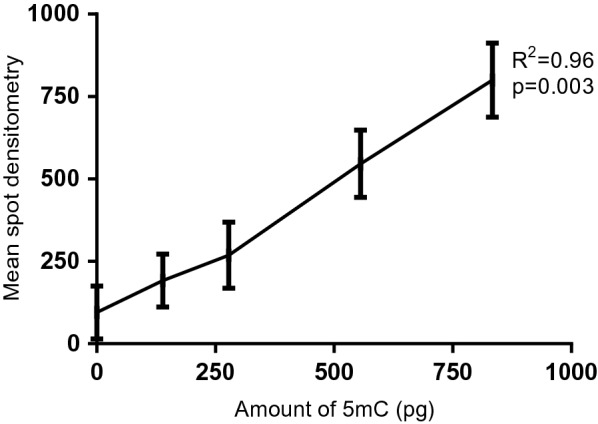
Linearity of the mean spot densitometry of the HeLa cells obtained from dot blot assay and the amount of 5mC in pg in each sample. Each ng of HeLa cells contains 4.63 pg of 5mC, five points shown in the graphic correspond to 0, 30, 60, 120 and 180 ng of HeLa DNA

In this work, we used a low-cost immunological fluorescence-based method to carry out a large sample screening to measure 5mC levels in the genome of the mollusk *B. glabrata* with an antibody against 5mC. A standard curve was generated with different concentrations of DNA from HeLa cells. We found a linear positive correlation between the densitometry measure of each HeLa cell’s DNA spots and the 5mC amount (Fig. 3, R^2^ = 0.96, *p* = 0.003).

### Flv1 decreased DNA methylation in the exposed generation and dot blot and ELISA-based Kit shows equivalent results

**Fig. 4:**
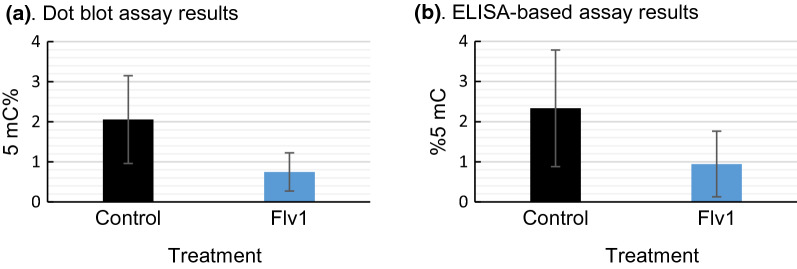
**a** 5mC% measures obtained by the dot blot method. **b** 5mC% measures obtained by the ELISA-based assay

Control and Flv1-treated *B. glabrata* snails (*n* = 15 per group) were used to measure their 5mC% by our dot blot method, and simultaneously, we measured the 5mC% with a commercial ELISA-based Kit. We found that there was significant difference between control and Flv1-exposed snails in the measures obtained by dot blot assay (*t* = 5.88, *df* = 32.70, *p* = 1.389e-06) and the same was found with ELISA-based commercial kit (*t* = 3.98, *df* = 34.53, *p* = 0.003273). Furthermore, the values obtained after normalization were similar between control samples in both methods, 2.05 ± 0.74 and 2.3 ± 1.4, respectively, and for Flv1-treated samples, they showed a 5mC% of 0.75 ± 0.47 with dot blot assay and 0.94 ± 0.81 with ELISA-based kit (Fig. 4).

Our results showed that dot blot assay delivered comparable results to established ELISA techniques. Dot blot assay allowed medium high throughput (about 200 samples per day) at low cost (about 3€ per sample compared to 19 € per sample with ELISA Kit) and required small amounts of DNA (30–100 ng). Furthermore, we found with both methods that Flv1 decreases the global 5mC% in the treated snails.

### Flv1 influences DNA methylation in vivo in the offspring of treated snails

**Fig. 5.**
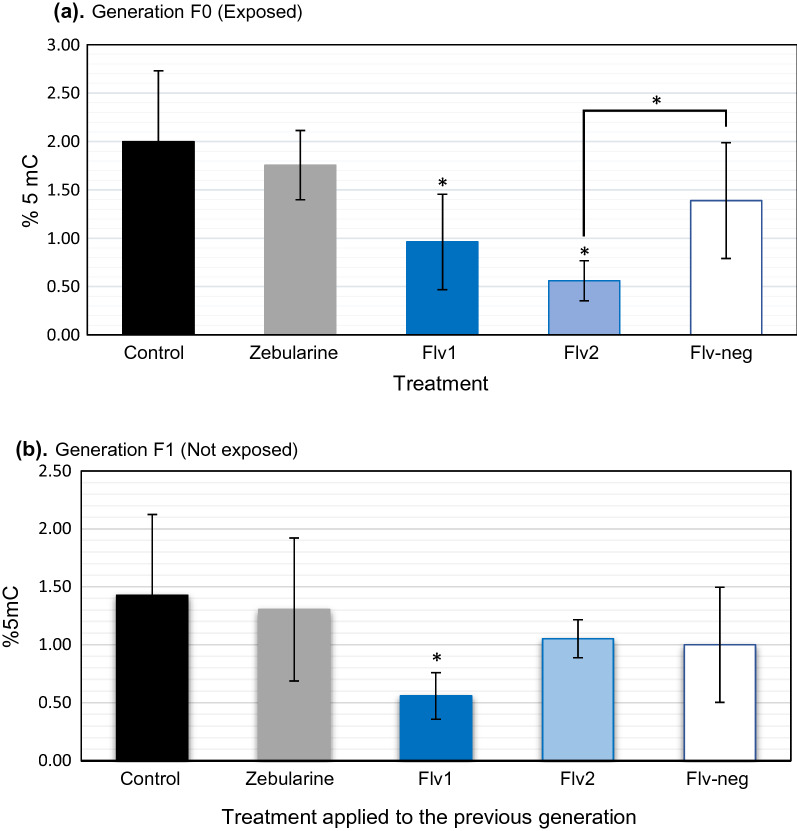
5mC% of *B. glabrata* snails upon DNMTi treatments at a concentration of 10 µM, error bars represent SD, *n* = 30 per treatment. **a** 5mC% in the F0 generation (exposed), black bar for control, gray bar for zebularine, blue bars represent flavanone inhibitors (blue bar for Flv1 and light blue bar for Flv2) and the white bar with blue outline represent the inactive Flv-neg. **b** 5mC% in the offspring of the exposed snails. Compounds are the ones used in F0. Significant differences are marked as * for *p* < 0.005. 5mC ng was normalized to the 5mC global percentage present in the genome of *B. glabrata*

Using dot blot method [56], we found significant differences in the 5mC% between treatments (Kruskal–Wallis test, *p* < 0.001). In the F0 generation, zebularine did not produce a statistically significant difference in 5mC% compared to the control group (Dunn’s test *p* > 0.9). However, 5mC% was significantly different in the groups treated with Flv1 (*p* < 0.001) and Flv2 (p < 0.001) compared to control group. The reduction in 5mC% between control (2%) and Flv1 (0.96%) was twofold (Fig. 5a). Unlike Flv1, Flv2 showed a significant difference compared to inactive flavanone Flv-neg (*p* = 0.005). In F1 generation, offspring snails of the Flv1-exposed generation presented a significantly lower 5mC% (*p* < 0.001) than the control group (Fig. 5b).

### DNMTi influence fecundity and morphometric traits

**Fig. 6:**
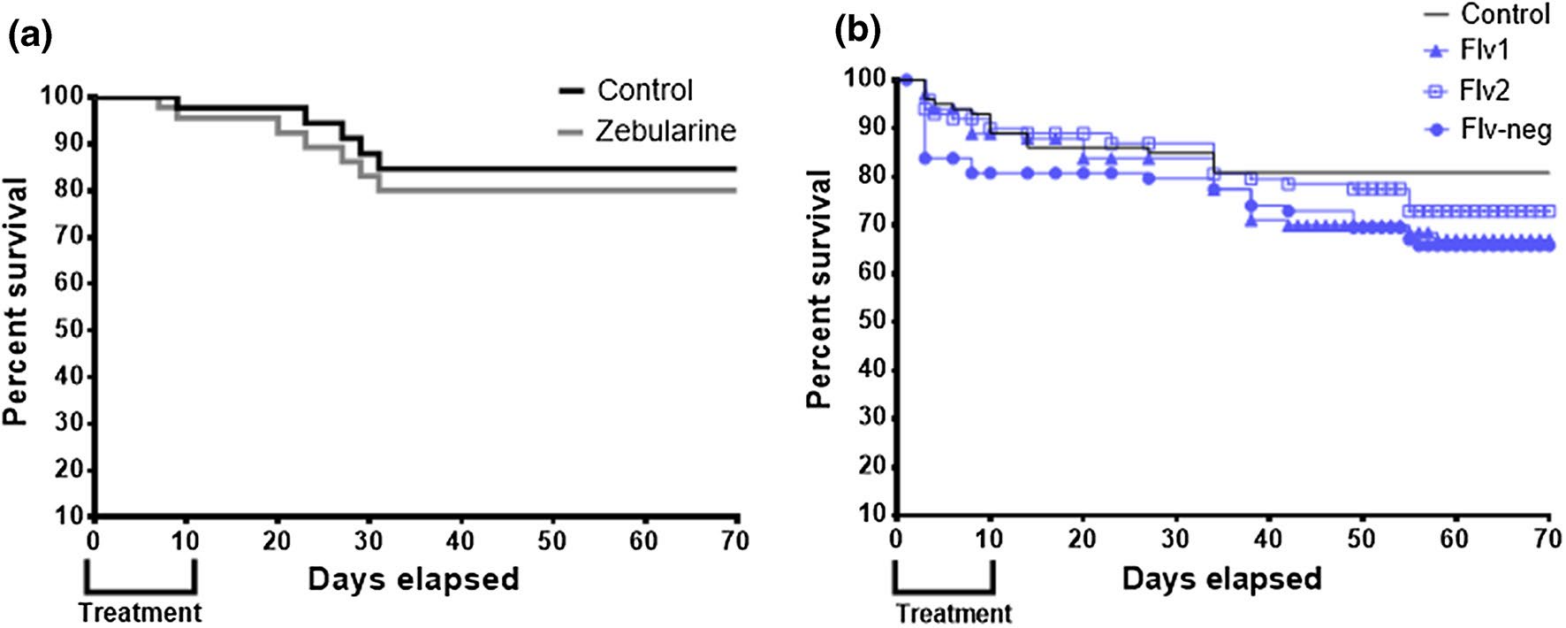
Kaplan–Meier survival curves upon treatment with the two types of DNMTi. **a** Cytidine-analogue zebularine (gray line). **b** Flavanones Flv1 (blue line with triangles) and Flv2 (blue line with squares) and their inactive derivative Flv-neg (blue line with circles)

Since our findings had clearly indicated that Flv1 had an in vivo and in vitro methylating inhibition activity, i.e., probably due to a direct effect on the DNMTs, we wondered if this epimutagenic activity had phenotypic consequences. Therefore, we measured survival, fecundity, and morphometric traits in DNMTi-treated snails and controls. We used as pharmacological reference molecule zebularine. Zebularine induced the lowest mortality with no significant difference compared to control group (Mantel-Cox test *χ*2 = 0.3, *p* = 0.56). This compound followed a similar trend as the control and the snail final survival rate reached 80% compared to 84% in the control (Fig. 6a). The mollusks treated with Flv1, Flv2 and inactive Flv-neg had a survival rate of 68%, 73%, and 69%, respectively (Fig. 6b), and none of these rates were statistically different compared to the control group (*χ*2 = 3.5, *p* = 0.06 for Flv1, *χ*2 = 1.16, *p* = 0.2 for Flv2 and *χ*^2^ = 5.9, *p* = 0.1 for Flv-neg).

The fecundity of snails was affected by the treatment with Flv2 and zebularine. Mollusks treated with Flv2 presented a low number of offspring (*n* = 20) and it was significantly different compared to control group (*p* = 0.004). Mollusks treated with zebularine presented a significantly lower offspring number than the control group (Fisher’s exact test, *p* < 0.0001) and the number of eggs was very high compared to the other treatments (Table 1). The treatments with Flv1 and Flv-neg showed no significant difference in the number of offspring against control group.

**Table 1:** Contingency table of fecundity of the snails exposed to different DNMTi

	Control	Flv1	Flv2	Flv-neg	Zebularine
Total number of laid eggs	191	183	199	188	326
Number of non-hatched eggs	152	147	179	147	301
Number of offspring	39	36	20*	41	25**

Total number of laid eggs (first row), number of non-hatched eggs (second row) and number of offspring snails (third row). Fisher’s exact test was applied, significant differences with control group are marked with * for *p* < 0.005 and ** for *p* < 0.0005

**Fig. 7:**
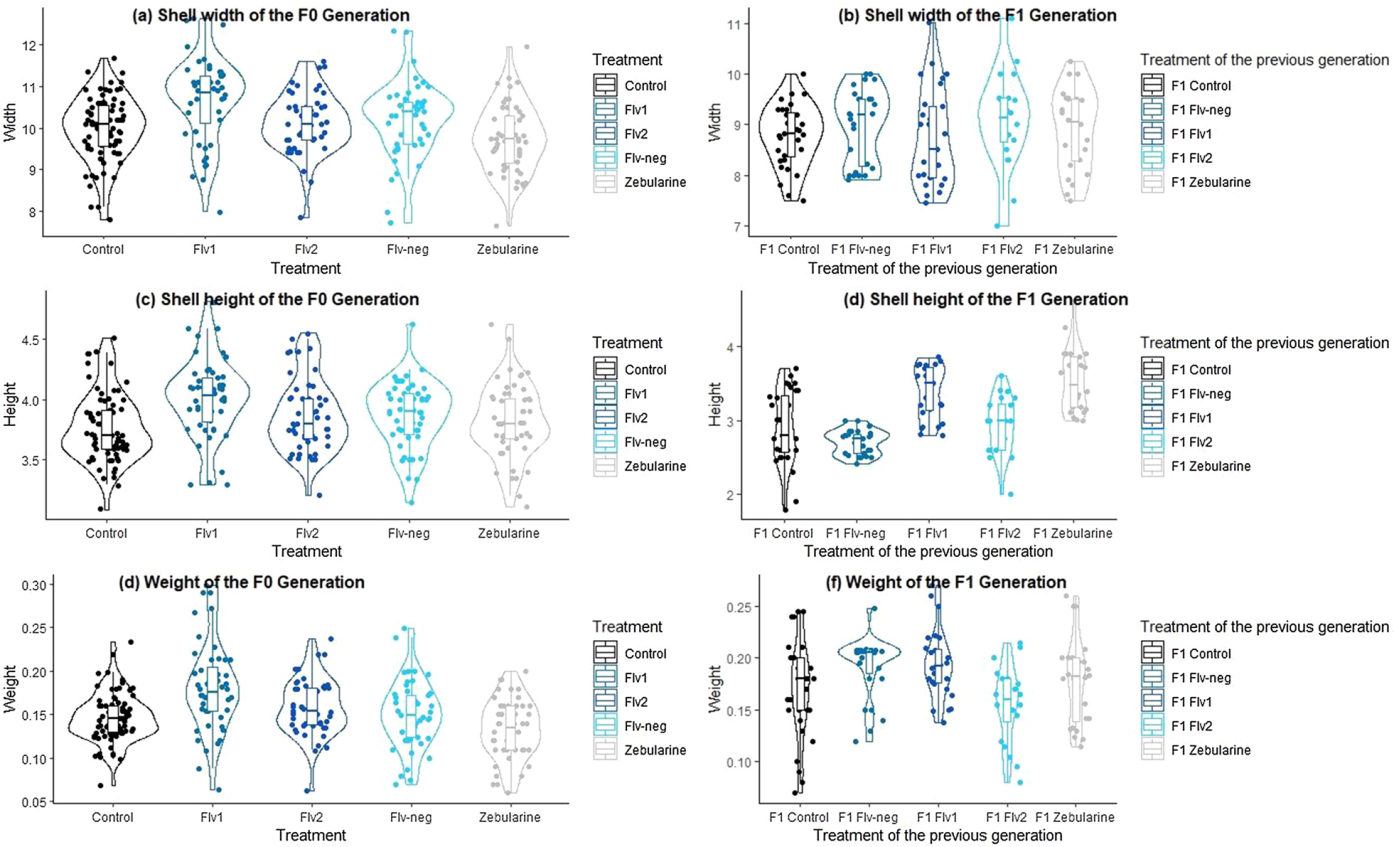
Violin plots of the morphometric traits between all treatments in both generations. **a** Shell width of the F0 generation, **b** shell width of the F1 generation, **c** shell height of the F0 generation, **d** shell height of the F1 generation, **e** weight of the F0 generation and **d** weight of the F1 generation. Violin plots showed kernel density estimations and a box indicating the interquartile range

To compare the variation of morphometric traits induced by DNMTi treatment in the F0 generation and in its respective offspring, we performed One-way ANOVA test, Welch’s ANOVA or Kruskal–Wallis test per morphometric trait (according to assumptions met). We found significant differences between the treatments of the F0 generation in the shell width (*p* = 4.001e-06), the shell height (*p* = 5e-04) and mollusk’s weight (*p* = 6.046e-07), and multiple pairwise comparison between treatments indicated that the difference between control and Flv1 treatment was significant for shell width (*p* = 0.00018), shell height (*p* = 0.0001286) and mollusk’s weight (*p* = 6.0e-05). In F1 generation, we found significant differences in shell height (*p* = 1.37e-13) and mollusk’s weight (*p* = 0.00277) between treatments, and multiple pairwise-comparisons, indicated that the difference between the offspring of the snails treated to Flv1 were significantly different to the offspring of controls for the shell height (*p* = 7.8e -05). Significant pairwise differences in the shell height were also observed between the offspring of controls and the offspring of snails treated to zebularine (*p* = 1.0e-05). Pairwise differences between flavanone compound were found for the mollusk’s weight in F1 generation, Flv2 was significantly different to Flv1 and Flv-neg (p = 0.006 and p = 0.01, respectively). To visualize the variation of distributions between treatments we performed violin plots (Fig. 7).

### Reduced representation of bisulfite-sequencing epiGBS reduces sequencing effort roughly 10 × and allows for reliable evaluation of global 5mC level and identification of differentially methylated sites and regions

To obtain a clearer picture of where hypomethylation occurred in the epigenomes of the DNMTi exposed populations and their offspring, we adapted a reduced representation technique that was originally developed for mosaic methylation of plants: epigenotyping by sequencing (epiGBS). Since it was only the second time epiGBS was used on mollusks, we first had to make sure that it delivered reliable results here. We used our previously obtained WGBS data [1] to compare the WGBS to epiGBS results on *B. glabrata*. We reanalyzed the WGBS data with updated pipeline analysis and generated a new reference methylome of *B. glabrata*. Using the BSMAP Mapper, 46.2% of reads mapped unambiguously to the *B. glabrata* reference genome. Paired-end sequencing of the 32 pooled epiGBS libraries (8 per treatment) resulted in a total of 140,751,495 filtered and demultiplexed reads. After quality control and alignment, an average of 34% of unique reads per sample mapped to the *B. glabrata* reference genome using BSMAP Mapper (Additional file 1: Table S1). After methylation calling, 6 samples per treatment with CpG sites covered by ≥ 8 reads were retained for further analysis, the removed samples showed very low number of CpG sites (< 4200). After filtering, we obtained an average of 47,715 ± 31,774 methylated CpG methylation positions per sample (Additional file 1: Table S1).

**Fig. 8:**
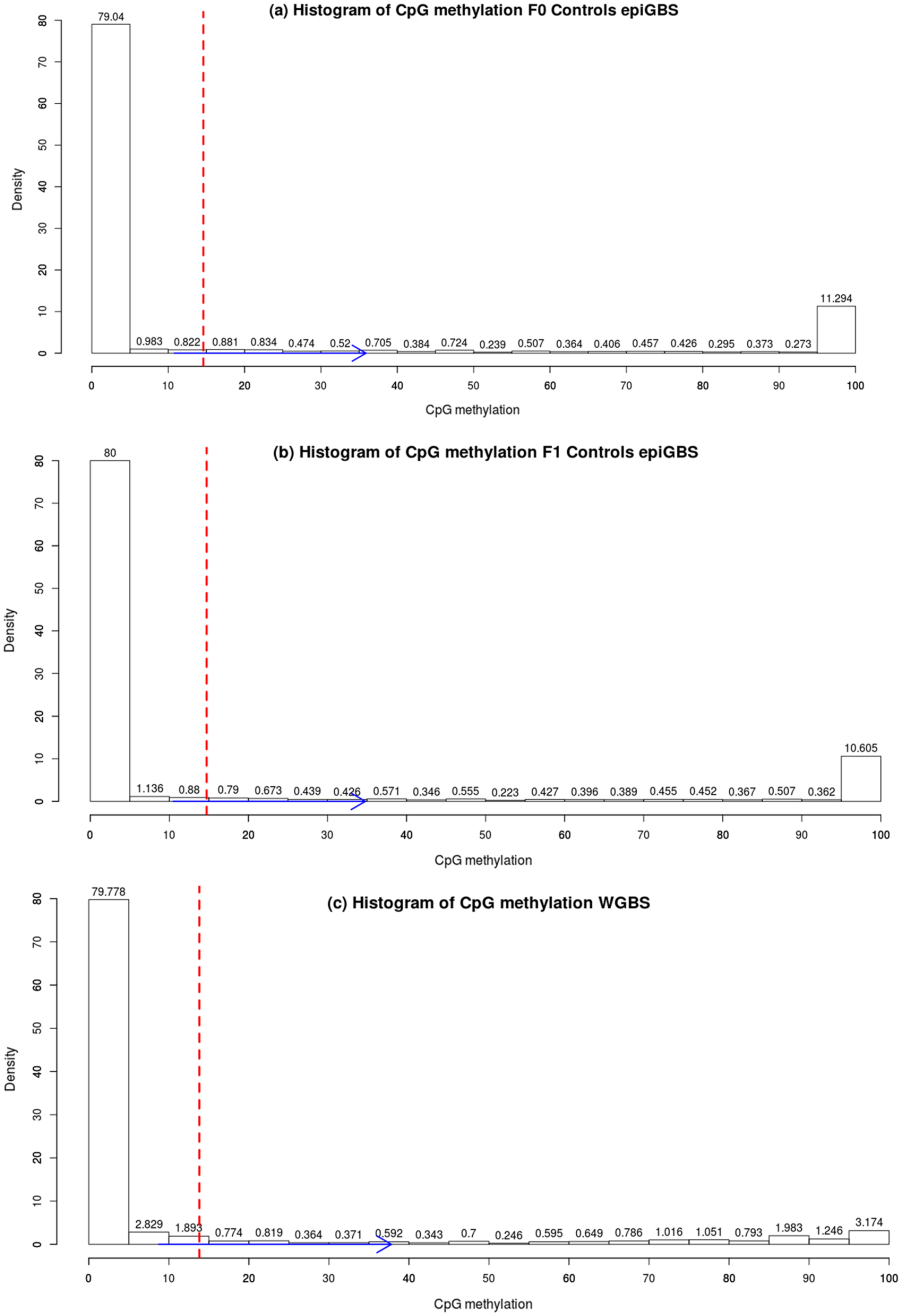
Histograms of CpG methylation distribution, **a** histogram of F0-control epiGBS libraries, **b** histogram of F1-control epiGBS libraries and **c** histogram of WGBS library. The abscissa represents the CpG methylation % (0–100) and the ordinate showed the density of CpG positions. The dashed red line indicates the mean CpG methylation value, and the blue arrow indicates the confidence interval (CI) of 95%

To analyze the distribution of methylated CpG over the entire genome, we represented its frequency distribution. We found a characteristic distribution of two peaks for both WGBS and epiGBS indicating the majority of the CpG sites being either unmethylated or completely methylated, as expected for a species that displays a mosaic distribution type of DNA methylation pattern (Fig. 8a–c). The peak of methylated CpG sites was higher in the epiGBS-sequencing results compared to WGBS, but mean CpG methylation values and confidence interval (CI) of 95% were highly similar in both methods (Fig. 8a–c).

A direct comparison was done to examine the data obtained for CpG methylation from epiGBS library versus WGBS (Additional file 1: Table S1). We chose the best covered control samples from each generation of epiGBS libraries to compare them with WGBS. WGBS data had a higher mapping efficiency than epiGBS (46.2% compared to 32.8%). The number of CpG sites with a minimum read coverage of 8× was of 34,646 and 63,892 for epiGBS libraries and 4,061,906 for WGBS. epiGBS represents 0.8% (epiGBS F0) and 1.6% (epiGBS F1) of the CpG sites covered by WGBS. However, the average levels of CpG methylation percentage were very similar between both the methods (Table 2).

**Table 2:** Mapping efficiencies, CpG coverage and average genome-wide methylation levels resulting from epiGBS and WGBS libraries

Samples	Mapping efficiency%	Sequence reads	Total no. CpGs	CpGs ≥ 8 × coverage	Methylated CpG sites	Methylated CpG%
epiGBS F0	34.3	707 010	95 369	34 646	7 621	22.0
epiGBS F1	34.5	1 299 293	180 852	63 892	13 982	21.9
WGBS	46.5	152 842 929	17 493 207	4 061 906	855 624	21.1

**Fig. 9:**
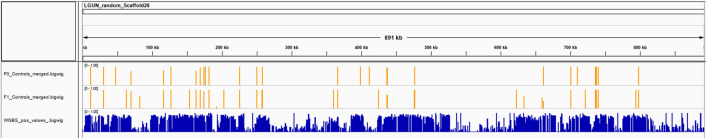
Screenshot of IGV of the region LGUN_random_Scaffold28: 1–800 Kb, that showed the regions covered by epiGBS libraries (in yellow) versus the regions covered by WGBS library (blue)

To evaluate concordance of epiGBS and WGBS, a correlation was done with the methylation values of CpG positions covered by both methods. A high correlation was found between WGBS and epiGBS, Spearman correlation, *R* = 0.74, *p* < 2.2e^−16^. We also visualized the CpG methylation profile of epiGBS samples compared to WGBS in IGV in a wide-ranging Scaffold. Visual inspection showed that both epiGBS libraries of F0 and F1-controls have similar methylation profiles (Fig. 9 yellow bars) while, naturally, epiGBS results represent a small fraction of the information found with WGBS (Fig. 9, blue bars).

**Fig. 10.**
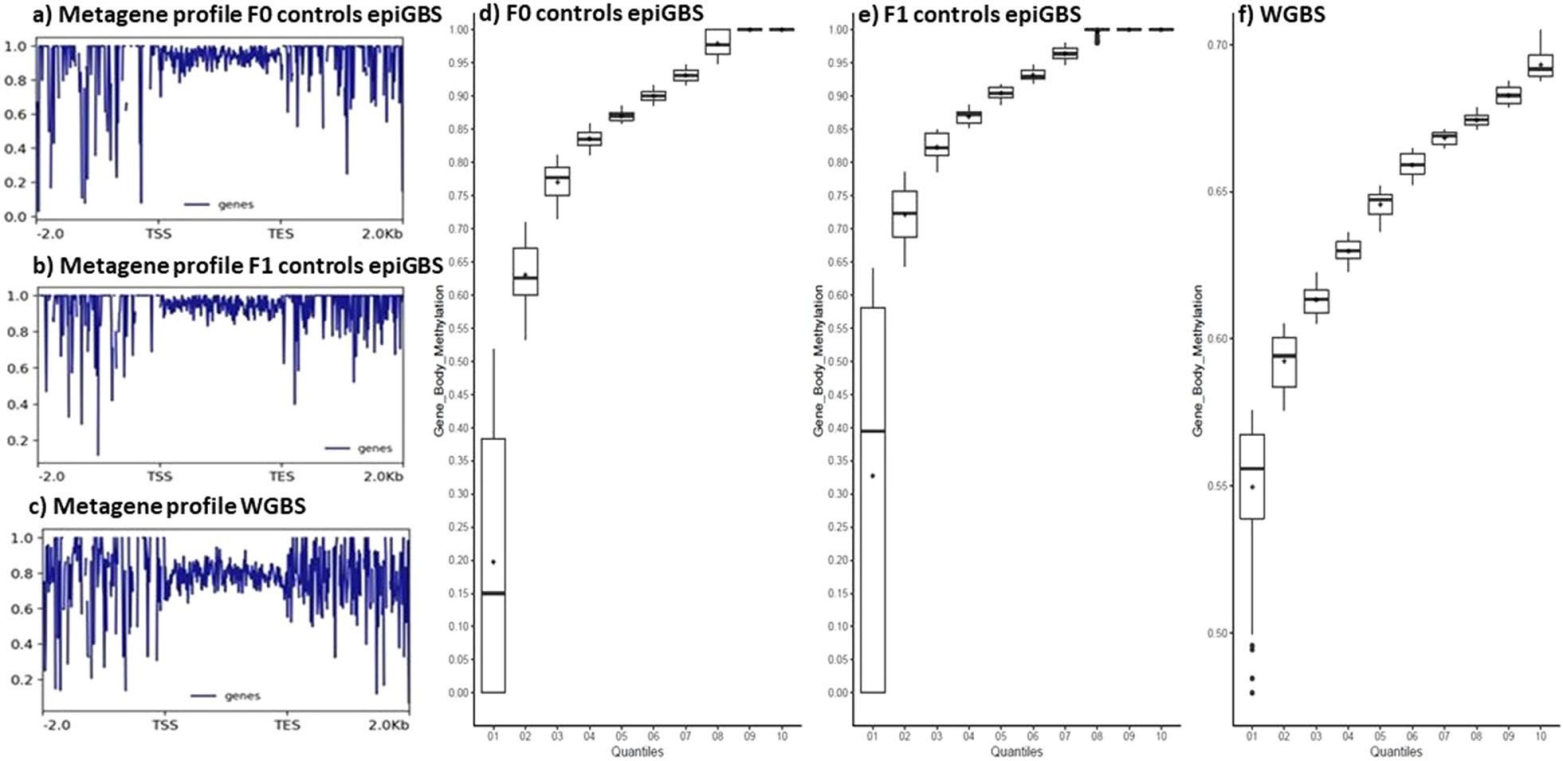
**a** Metagene profile of CpG methylation ratio of F0-control epiGBS libraries, Y-axis shows CpG methylation ratio profile across the bodies of genes as average methylation ratio overlapping a 50 bp bin and x-axis − 2.0 kb indicates the upstream 2000 bp of TSS, and 2.0 kb indicates the downstream 2000 bp of TES. All gene length arbitrarily scaled to 2 kb **b** metagene profiles of CpG methylation of F1-control epiGBS libraries and **c** WGBS library. Quantiles (deciles) distribution of Gene body methylation of **d** F0-control epiGBS **e** F1-control epiGBS and **f** WGBS. **d–f** Y-axis shows CpG methylation ratio profile across the bodies of genes and x-axis shows deciles (cut points dividing the range of probability distribution into ten continuous intervals) of epiGBS and WGBS libraries.

We then produced the CpG methylation metagene profiles across gene bodies from 2 kb upstream of the transcription start sites (TSS) and 2 kb downstream of the transcription end sites (TES). The CpG sites used for these profiles were those covered by both methods (14,340 CpG sites). We found that CpG methylation levels remained a plateau after TSS and along the gene bodies and then showed a high range of methylation before TSS and after TES in both methods. The range of GBM levels was different in epiGBS libraries (0.9–1) (Fig. 10a, b) than in WGBS (0.7–0.9) (Fig. 10c).

We chose the 14,340 CpG sites within gene bodies (GBM) covered by epiGBS and WGBS libraries to calculate quantile distribution. The quantile distribution of GBM was slightly different between epiGBS and WGBS. In the epiGBS libraries of F0- and F1-controls, the quantile with the highest range is the first one and comprises CpG values of 0.15–0.80 and 0.47–0.85 (Fig. 10d, e). Otherwise, in the WGBS library, the first quantile comprises values from 0.07 to 0.58 (Fig. 10f).

The global distribution of CpG methylation sites displayed a two-peak histogram in all epiGBS samples, with most of the CpG sites being either unmethylated or completely methylated. In summary, epiGBS mirrors WGBS on a global scale but has necessarily a lower resolution (at our sequencing depth about 1% of the CpG sites were captured) and it also has a slight bias towards methylated regions of the epigenome.

### epiGBS corroborates hypomethylation in the offspring of snails treated with Flv1 inhibitor

We considered epiGBS a reliable method that allows for epigenome-wide analysis of DNA methylation changes in populations at reasonable costs and we used it to capture regional methylation differences in Flv1-treated samples. The mean percentage of CpG methylation was 15.8 ± 0.8% in control snails and 13.5 ± 0.6% in Flv1-exposed snails, 13.5 ± 0.3% in offspring of control snails and 13.1 ± 0.1% in the offspring of Flv1-exposed snails. There was significant difference in global percentage of CpG methylation between control and Flv1-exposed snails (*t* = 6.0, *df* = 9.4, *p* = 0.0001) and significant difference was also found in their offspring (*t* = 3.0, *df* = 6.0, *p* = 0.023).

**Fig. 11:**
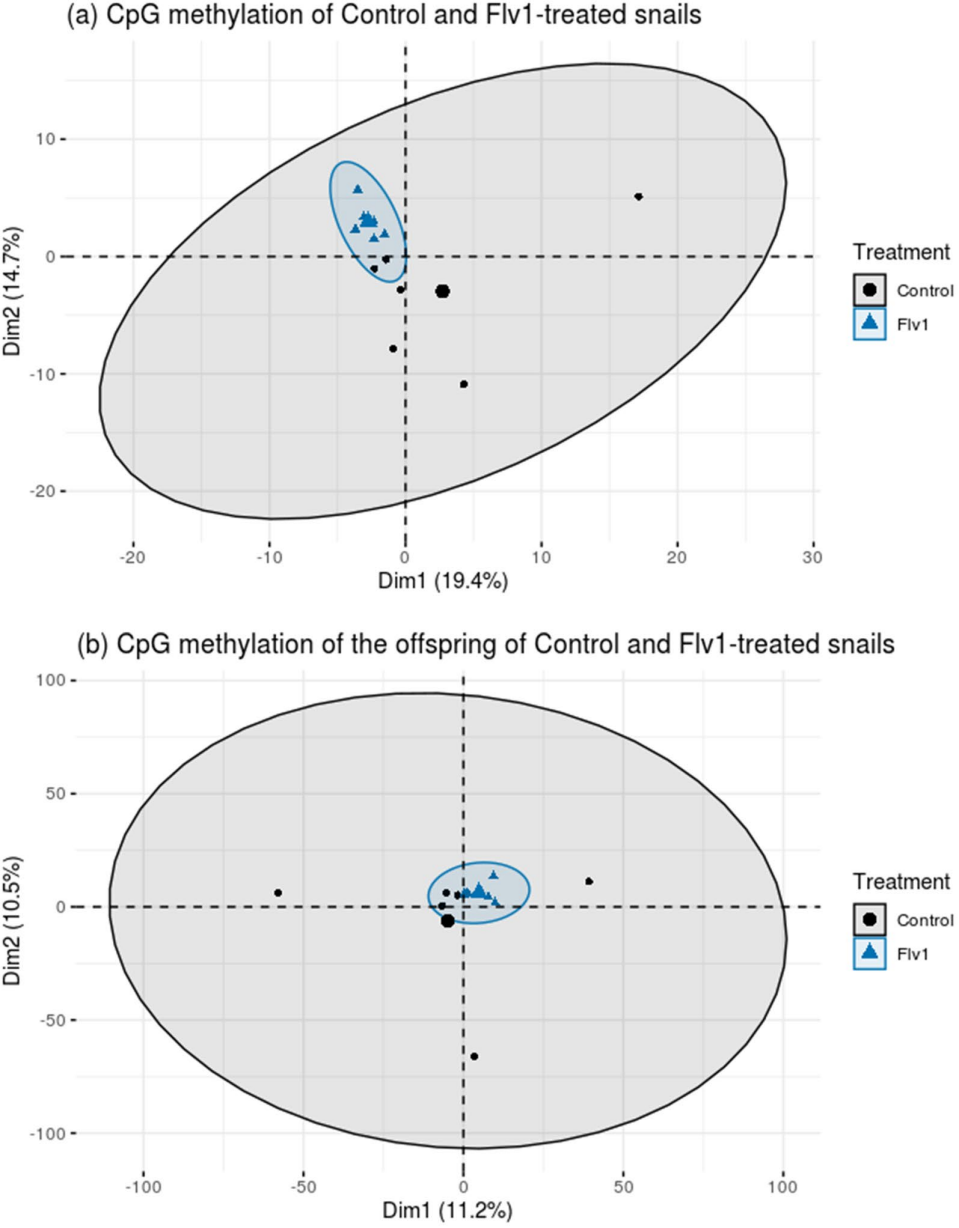
PCA of CpG methylation of the Flv1-treated and control groups in F0 generation (**a**) and in its offspring (**b**). The ellipses represent the 95% confidence interval

PCA analysis of CpG methylation was performed on controls and Flv1-treated samples (Fig. 11). Interestingly, Flv1-treated samples clustered tightly, while control samples were spread out (Fig. 11a). PCA analysis of CpG methylation in F1 generation showed the same tendency, F1-Flv1 samples were grouped, and F1-control samples dispersed (Fig. 11b). PCA of both generations displayed the same pattern, indicating an impact in the CpG methylation at the genome-wide level and a decrease of CpG methylation variability/diversity in both generations.

### Five out of 32 Flv1-induced hypomethylated DMRs are present in 2 consecutive generations

Differential Methylated CpG sites (DMCs) were analyzed between control and Flv1 treatment in both generations. We found 44 DMCs in the F0 generation between control and Flv1-exposed samples, comprising 31 hypomethylated and 13 hypermethylated with the strict methylkit parameters. With the adapted parameters for small sample sizes and weak methylation effects described in [80], we found 245 DMCs, 153 hypomethylated and 92 hypermethylated (Table 3). Interestingly, these DMCs are not isolated and rather concentrated in specific regions (within 1 Kb), indicating the presence of differential methylated regions (DMRs). Considering DMR with ≥ 2 DMCs, the 44 DMCs found with the strict methylkit parameters comprise 10 DMRs and the 245 DMCs found with the adapted methylkit parameters comprise 32 DMRs (Additional file 1: Tables S2, S4).

**Table 3:** DMCs and DMRs in Flv1-treated and control group for each generation

Generation	*q* value = 0.05, difference = 15% DMCs	*q* value = 1.0, difference = 4% DMCs	*q* value = 0.05, difference = 15% Hypomethylated DMCs	*q* value = 1.0, difference = 4% Hypomethylated DMCs	*q* value = 0.05, difference = 15% Hypermethylated DMCs	*q* value = 1.0, difference = 4% Hypermethylated DMCs
F0	44	245	31	153	13	92
F1	332	1900	207	1153	125	747
F0 and F1	6	41	6	23	0	18
Generation	*q* value = 0.05, difference = 15% DMRs	*q* value = 1, difference = 4% DMRs	*q* value = 0.0.5, difference = 15% Hypomethylated DMRs	*q* value = 0.1, difference = 4% Hypomethylated DMRs	*q* value = 0.0.5, difference = 15% Hypermethylated DMRs	*q* value = 0.1, difference = 4% Hypermethylated DMRs
F0	10	32	6	17	4	15
F1	51	345	38	200	13	145
F0 and F1	1	7	1	5	0	2

Two setting parameters were used to calculate the DMCs, the strict parameters with a *q* value < 0.05 and a cutoff of 15% methylation difference and the adapted parameters with a *q* value < 1 and a cutoff of 4%

In F1 generation, 332 DMCs were found between F1-control and F1-Flv1 samples, 207 hypomethylated and 125 hypermethylated (Additional file 1: Table S3) with the strict methylkit parameters, and 1900 DMCs were found between F1-control and F1-Flv1 samples with the adapted methylkit parameters, from which 1153 were hypomethylated and 747 hypermethylated (Additional file 1: Table S5). The majority of hypomethylated DMCs with both parameters demonstrates a hypomethylated genome-wide effect. The number of DMCs in the F1 generation was higher than in the generation F0.

**Fig. 12:**
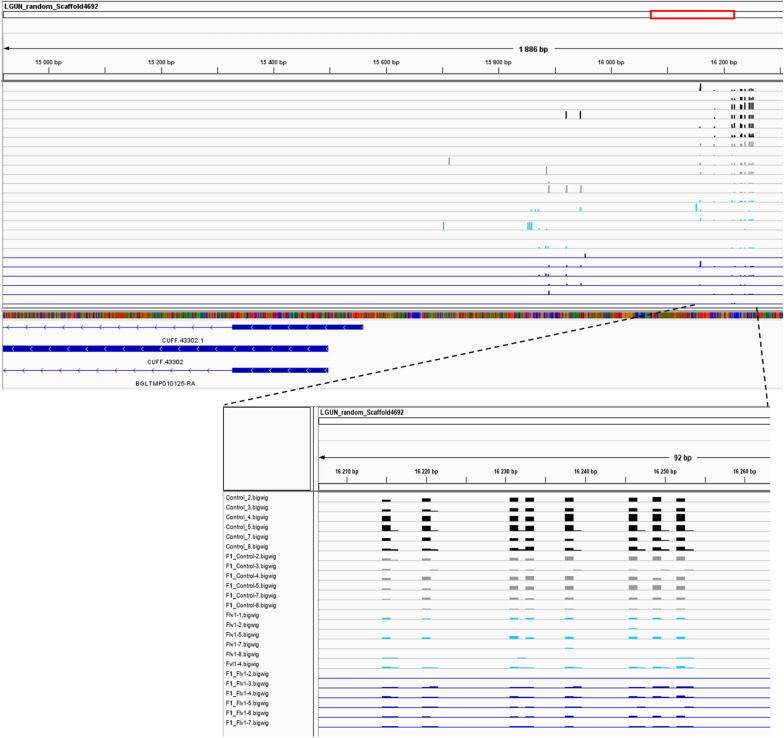
IGV screenshot of the LGUN_random_scaffold4962:16175-16266 of *B. glabrata* genome assembly (GCA_000457365.1). Each bar indicates the position of a methylated CpG site for the different samples: F0-control F0 (black), F1-control (gray), Flv1-treated samples (light blue) and its offspring (dark blue). Hypomethylated DMCs have been detected in this region and are in common between F0 and F1 generations

Using adapted methylkit parameters, we found 41 DMCs common between both generations, being 23 hypomethylated and 18 hypermethylated, the hypomethylated DMCs are concentrated in 5 DMRs and the hypermethylated DMCs in 2 DMRs (Additional file 1: Table S7). The context of each DMR found in common between each generation was examined visually in the Integrative Genomics Viewer (IGV) using the *B. glabrata* genome (Assembly GCA_000457365.1) and the reference transcriptome for annotation. The two hypermethylated DMRs found in common in both generations are in an intergenic context, in the case of hypomethylated DMRs, four are in the intergenic context and one at the putative promoter region of the transcript BGLTMP010125 (Fig. 12).

### The BGLTMP010125 gene is hypomethylated by Flv1 and shows decreased transcription

**Fig. 13.**
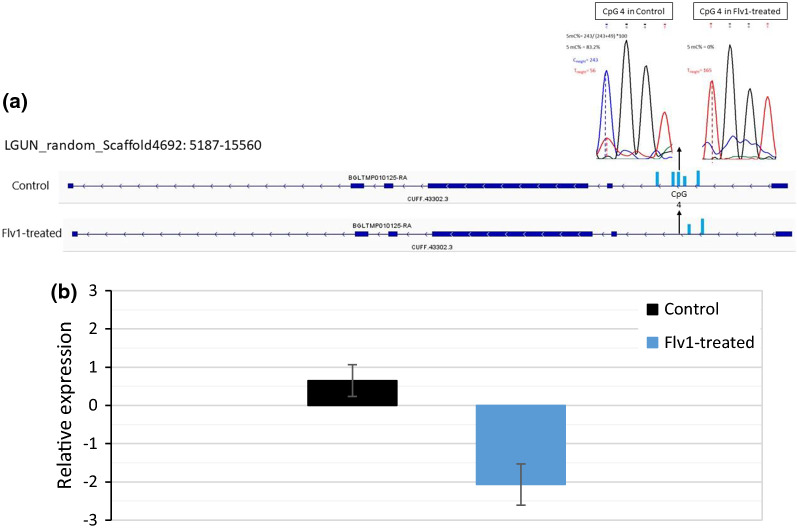
**a** Position and level of methylation of the five CpG positions, which has been studied by TBS within the first intron of the transcript BGLTMP010125-RA. **b** Relative expression of the transcript BGLTMP010125-RA compared to two housekeeping genes (28S and α-Tubulin), the ordinate shows the logarithm of the values obtained with the 2^−ΔΔCT^ method

One of the identified DMR was particularly intriguing. This DMR that was hypomethylated in Flv1-treated snails and in their offspring was close to transcript BGLTMP010125. However, no CpG sites within the BGLTMP010125 were detected by our epiGBS approach so that we could not evaluate gene body methylation (GBM) by this method. We, therefore, decided to apply targeted bisulfite sequencing (TBS). We chose a region in the first intron of the transcript, roughly 2 kb upstream of the DMR and spanning 9 CpG to further explore the relationship between GBM and gene expression. Our TBS results showed that control snails had five methylated CpG sites in the targeted region of the transcript BGLTMP010125-RA and that the Flv1-treated snails showed a decrease of the 5mC level in three of the five CpG sites (Table 4), in the CpG 4 of the control snail 6, the decreased of CpG methylation percentage was from 83.2 to 0%. Mean methylation over the 9 CpGs analyzed was significantly lower in Flv1-treated snails (*t* = 10.58, *df* = 8.18, *p* = 4.673e−06) than in controls (Fig. 13a) and the transcript was significantly lower in Flv1-treated samples compared to controls (*t* = 6.53, *df* = 10.02, *p* = 6.477e−05) (Fig. 13b).

**Table 4:** 5mC% per CpG sites in the bisulfite-converted sequence of the transcript BGLTMP010125

CpG sites	*CpG 1*	*CpG 2*	*CpG 3*	*CpG 4*	*CpG 5*	*CpG 6*	*CpG 7*	*CpG 8*	*CpG 9*
Position on Contig LGUN_random_Scaffold4692	*13866*	*13976*	*14024*	*14042*	*14059*	*14061*	*14317*	*14331*	*14343*
Control 1	100	0	100	100	0	63.4	0	100	0
Control 2	88.4	0	94.2	83.3	0	62.9	0	95.2	0
Control 3	100	0	97.8	100	0	87.8	0	100	0
Control 4	100	0	100	100	0	40	0	100	0
Control 5	100	0	100	83.1	0	73.4	0	100	0
Control 6	100	0	100	100	0	60	0	100	0
Control 7	89.5	0	95.3	100	0	81.1	0	100	0
Control 8	100	0	100	83.2	0	63.0	0	100	0
Flavanone 1	0	0	0	0	0	79.6	0	76.0	0
Flavanone 2	0		0	0		804		100	
Flavanone 3	0	0	0	0	0	54.3	0	93.7	0
Flavanone 4		0		0	0	59.4	0	100	0
Flavanone 5	0	0	0	0	0	60	0	100	0
Flavanone 6	0	0	0	0	0	71.9	0	100	0
Flavanone 7	0	0	0	0	0	60	0	100	0
Flavanone 8	0	0	0	0	0	68.4	0	100	0
Control mean	97.2	0	98.4	93.7	0	66.4	0	99.4	0
Flavanone mean	0	0	0	0	0	66.7	0	96.2	0

### Flv1 reduces global 5mC in other mollusks

**Fig. 14.**
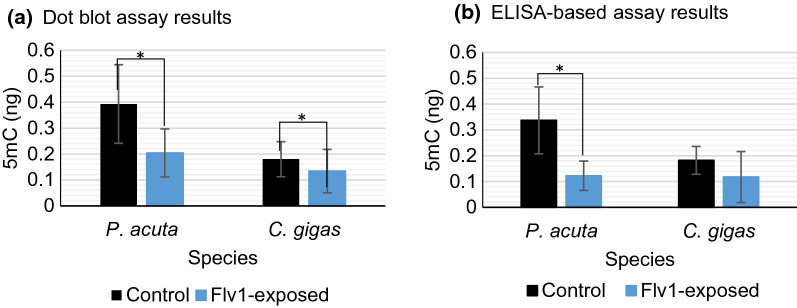
**a** 5mC% measures obtained by the dot blot method for *P. acuta* and *C. gigas*. **b** 5mC% measures obtained by the ELISA-based assay. The bars represent the 5mC (ng) mean, the error bars represent the standard deviation (SD), *n* = 30 per group per specie for dot blot and *n* = 10 for ELISA. Significant differences between treatment and control are marked as * for p < 0.05

Since Flv1 showed efficiency as DNMTi in *B. glabrata*, and it mostly affected the offspring, we wondered if it would be active also in other mollusk species and we tested Flv1 compound in the juvenile stages of *P. acuta* and *C. gigas*. The dot blot results (Fig. 14a) displayed that Flv1-exposed *P. acuta* snails have a significantly decrease of the 5mC (ng) concentration compared to controls (*t* = 5.90, *df* = 52.23, *p* = 2.76 e-07). For *C. gigas*, the decrease in 5mC (ng) by the Flv1 treatment was also significantly different compared to control (*t* = 2.18, *df* = 47.946, *p* = 0.0342). The ELISA results (Fig. 14b) showed that the Flv1 compound decreased significantly the 5mC concentration (ng) in *P. acuta* snails compared to controls (*t* = 4.80, *df* = 12.33, *p* = 0.0004), and for *C. gigas*, we did not find a significantly decrease of the 5mC (ng) (*t* = 1.48, *df* = 12.11, *p* = 0.16) in ELISA-based results but we found a tendency to decrease.

## Discussion

An extension of the concept of inheritance system includes the genotype, the epigenotype, the heritable cytoplasmic elements and the microbiome that interacts with the environment to shape and transmit the phenotype [17]. The epigenotype and the microbiome can be altered by environmental factors and these modifications can be inherited, at least in some systems, through generations, potentially facilitating genetic adaptation. One of the most-studied epigenetic mark is DNA methylation. It has been widely studied in vertebrates and plants but remains poorly understood in invertebrates, one of the largest phyla of invertebrates are mollusks, that include several species that are commercially, ecologically, and medically important. It was hypothesized that DNA methylation in mollusk can be a mechanism to produce phenotypic variation and potentially adaptation to new environments [69], but experimental proof is lacking. DNA methylation in mollusks is likely to be an important element of the inheritance system. One way to analyze its role is to expose the inheritance system to external perturbations that target specifically the DNA methylation, e.g., using DNMTi. Such specific inhibitors were synthetized to be used in human cell lines and they were applied to other phyla assuming they would have the same effect. This strategy already led to important advances in other species where treatments with the most used DNMTi, 5-AzaC, were correlated with demethylation and phenotypic changes [5, 31, 57]. Nevertheless, this drug has shown low response rates, low stability in aqueous solutions and a high toxicity [81]. New DNMTi have been developed to overcome these weaknesses [65]. The aim of this work was to find an efficient DNMTi for mollusks that (i) provoked minimal side effects and (ii) allowed the study of the DNA methylation contribution to phenotypic variability and the heritability of environmental DNA methylation changes.

We used here an antibody-based assay as a screening method of global 5mC% modifications. We determined a linear correlation between DNA amount and mean spot density in the dot blot assay, and we demonstrated that it showed comparable results to ELISA-based commercial kit but allowing the screening of a larger number of samples at a lower price (Figs. 3 and 4). Furthermore, we used epiGBS, a reduced representation bisulfite sequencing providing evidence that this method can be used to analyze genome-wide DNA methylation changes. This method allowed the analysis of DNA methylation changes at the nucleotide level of numerous replicates, that is a pre-requisite for ecological studies, at an affordable price and giving results that represent the same global pattern as WGBS, as shown by the high correlation found between the methylation ratios of the CpG positions covered by both methods (Spearman correlation, *R* = 0.74, *p* < 2.2e^−16^). We captured only 1% of the CpG sites found in the genome, however, epiGBS laboratory protocol and bioinformatics analysis are very flexible and can be further improved to obtain higher coverage [30].

### Zebularine is not suitable for DNA methylation modification in *B. glabrata*

Zebularine has been reported as an efficient DNMTi in vertebrates, especially in human cancer cell lines [77]. In this work, we evaluated its effect on the DNA methylation and phenotypic variation on the snail *B. glabrata*. We decided to use this drug as it is associated with lower cytotoxicity than the nucleoside analogs (5-AzaC and 5-Aza-deoxycytidine) due to a different mechanism of action and higher stability in aqueous media [14, 26]. Nevertheless, the decrease of global DNA methylation was not significant following zebularine treatment. Moreover, we observed an increase in the oviposition of snails treated with Zebularine (Table 2). This phenomenon was also observed in snails exposed to the parasite *S. mansoni* [79], where oviposition is increased during the first days of parasite exposure. This response may be a fecundity compensatory strategy for expected future suppression of egg-laying and it is caused by environmental stress and the toxicity of zebularine possibly triggered this response. Zebularine has demonstrated a transient hypomethylation effect in plants [8], we cannot exclude that the same could happen in *B. glabrata*, since we observed some phenotypic effects, such as in the fecundity, presenting the lowest percentage of hatching rate in the exposed generation and a significant difference between the shell height of the offspring treated with zebularine and the offspring of the control group, and a tendency to decrease in the global methylation level was also observed. In addition, zebularine is not a specific inhibitor of DNMTs, it also inhibits cytidine deaminase, an important enzyme in the biosynthesis of nucleotides, which can explain the phenotypic effects observed, nevertheless it could be a less efficient DNMTi as most of the compound can be sequestered by this enzyme, and therefore, lowering its effective concentration. This is concordant with other studies showing that, to have an effective inhibition of DNMTs, a high concentration of this compound was required (≥ 100 µM) [16].

### Flavanone-type inhibitor has no toxic effects and reduces 5mC level in exposed and offspring of exposed snails

As a nucleoside analogue, the mechanism of action of zebularine requires its incorporation into DNA after phosphorylation and its conversion to the deoxy-zebularine triphosphate. The new DNMTi tested in this work are non-nucleoside analogues that do not incorporate into DNA being potentially more specific to DNMTs [37]. We tested the Flv1 analogue and showed that Flv1 triggered a significant decrease on 5mC% in the F0-exposed generation and in the subsequent non-exposed F1 generation in *B. glabrata*. Moreover, we found significant differences in the global methylation level of juvenile mollusks of the species *P. acuta* and *C. gigas* exposed to the Flv1 compound. We further tested the stability of the flavanone compounds (3-halo-3-nitroflavanones) in freshwater and in sea saltwater that was used to raise our mollusks models. We found differences in the chemical stability of Flv1 between freshwater and sea salt water, the compound was ~ 3 times more stable in freshwater than it was in sea salt water (Fig. 1), this can explain the results for *C. gigas* (raised in seawater) where diminution of global DNA methylation was lower than in the freshwater snails *P. acuta* and *B. glabrata.* In addition, we demonstrated an in vitro DNMT inhibition activity of the Flv1 compound in a nuclear extract from *Bge* cells, the embryonic cell line of *B. glabrata*. This suggests that 5mC modulation triggered by Flv1, was most likely due to direct inhibition of enzymatic DNMT activity.

At the phenotypic level, no significant differences were found in the survival and fecundity between Flv1 and its negative analog (Flv-neg) against the control group. Moreover, Flv1 treatment triggered significant changes compared to controls in the morphometric traits, the snails treated with Flv1 present a significant higher shell width, shell height and mollusks weight against controls; this suggests that the inhibition of DNMT activity by Flv1 led to a significant change in the morphometric traits in the exposed generation. Similar results were found in *S. mansoni* [18], where we found significant differences in the body length of the parasite larvae between control group and group treated with the epimutagenic TSA, a histone deacetylase inhibitor. Both results showed that modification of epigenetic marks by specific drugs can have effects on the phenotype variability of organisms. Nevertheless, more work is needed to verify if epimutations at multiple loci are causing the observed phenotypic variability through post-transcriptional or gene expression changes or if phenotypic variability is independent of these induced epimutations.

The offspring of the mollusks treated with Flv1 were significantly higher in the shell height compared to the offspring of control snails. This suggests that the germline was possibly affected by the Flv1 inhibitor which affected the shell height of their offspring.

Flv2 showed a negative impact in the fecundity of treated snails and a significant decrease of the global 5mC in the treated snails that was significantly different compared to Flv-neg. Moreover, the offspring of the mollusks treated with Flv2 were significantly lower in their weight compared to the offspring of those treated with Flv1 and Flv-neg. These effects suggest a side effect of this compound that was probably due to its different chemical composition. Flv2 possesses a chlorine atom in the α-position of the carbonyl group and Flv1 possesses a bromine atom at the same position, which makes that the two compounds present different DNMT inhibition potency, cytotoxicity, and stability [65]. Flv1 induced a higher gene expression activity in KG1-cells, by decreasing promoter methylation compared to Flv2. Moreover, Flv1 triggered lower cytotoxicity compared to Flv2 in different cancer cell lines [65] and in our results, it showed lower stability in freshwater than Flv2. Therefore, Flv2 was more stable in freshwater but also more toxic against snails which explains its high effect in F0 generations and its subsequent compensatory effect in the next generation in the form of hypermethylation, similar results were found in DNA methylation levels of the TRL 1215 cell line after Cadmium exposure, which inhibits DNA methyltransferases as part of its toxicity and when exposure is prolonged it results in DNA hypermethylation [76]. Flv-neg was inactive in an in vitro inhibition assay against purified human DNMT3A-c [65].

We corroborated Flv1 hypomethylation effect in *B. glabrata* by high-throughput bisulfite sequencing with the epiGBS method, confirming that the average of overall percentage of CpG methylation was significantly lower in Flv1-exposed snails compared to controls and the same trend was found in its offspring. To find differentially methylated cytosines, we decided to analyze our data using two levels of stringency: (1) a strict and most stringent one filtered the DMC based on a *q* value < 0.05 and 15% of methylation difference that has been normally used in other mollusks species and has been defined based on studies of model species [2] and (2) an adapted method filtered the DMC based on a *q* value < 1 and 4% of methylation difference and has previously been applied to small sample sizes and allows to find small differential methylation effects [80]. A total of 44 DMCs were found in the Flv1-exposed snails compared to controls with the strict methylkit parameters and 245 DMCs with the adapted methylkit parameters. Its progeny showed 332 DMCs compared to F1-controls with the strict methylkit parameters and 1900 DMCs with the adapted methylkit parameters. The higher number of DMCs in the F1 generation might be due to an indirect exposure of the germline to the inhibitor. In mollusks, germ cells appear early in the embryonic development [55]. It has been demonstrated that exposure of the germline to DNMTi affects epigenetic programming in sperm and oocytes and are likely to affect outcomes and offspring development principally in vertebrates [66, 85].

Seven DMRs were observed in Flv1-exposed snails and in its progeny, five hypomethylated and two hypermethylated, demonstrating a multigenerational effect, resulting probably from a direct exposure of the germline to the inhibitor. Few examples of multigenerational effect have been reported in mollusks [24], one is our previous study in *C. gigas* showing that a parental herbicide exposure strongly affected the offspring DNA methylation pattern [70]. Another example was found in *P. acuta*, where exposure to prednisolone, a steroid hormone evacuated from hospital wastewater, negatively affected the phenotypic traits of the snail, exhibited multigenerational toxicity, and affected global DNA methylation of adult progeny [7].

One of the hypomethylated DMRs found in both *B. glabrata* generations mapped to the putative promoter region of transcript BGLTMP010125 coding for a thump domain-containing protein 3-like. A protein BLAST (blastp) with the amino acid sequence of this protein showed 66.4% of identity with the THUMP domain-containing protein 3-like of *Aplysia californica* (NCBI reference sequence XP_012941090) and 52.8% of identity with the THUMP domain protein 3 of the brachiopod *Lingula anatina* (XP_013378720.1), and these proteins are part of AdoMet_MTases superfamily, enzymes that use *S*-adenosyl-l-methionine (Adomet) as a substrate for methyltransferase, creating the product *S*-adenosyl-l-homocysteine. TBS in the first intron showed that the Flv1 inhibitor treatment decreased significantly the GBM level in this transcript. qPCR indicated reduced gene expression in Flv1-treated F0 generation. Interestingly, the gene impacted by the inhibitor is coding for a AdoMet-dependent methyltransferase whose decreased expression could have leverage effects on 5mC level at multiple loci by influencing AdoMet homeostasis. These results are in agreement with earlier results in the invertebrates *Nematostella vectensis* and *Bombyx mori* [87, 88], where a positive linear correlation was found between GBM and mRNA levels.

In conclusion, the compound Flv1 was sufficiently stable in freshwater for 4 h to trigger cytosine modifications that were transmitted through mitosis in the F0 generation and through meiosis to the F1 generation. Flv1 demonstrated to be a good candidate to perform multigenerational DNMTi experiments: it neither impacts fecundity nor survival and it induces seven DMRs found in two consecutive generations; moreover, it showed higher inhibition potency in the offspring of treated individuals probably due to a direct impact in the germline. Since DNMTs are a conserved family of cytosine methyltransferases and since we showed that Flv1 inhibitor is efficient in another two mollusk species *P. acuta* and *C. gigas*, we suggest that this new DNMTi could be used to pharmacologically modify 5mC level in mollusks, providing a tool to study the inheritance of 5mC environmental modifications in this taxon.

In neo-Darwinian theory, genetic variation is considered a pre-requisite for hereditary phenotypic variation and as the primary material of adaptation by natural selection. Nevertheless, it has been demonstrated that the epigenetic inheritance system allows the environmentally induced phenotypes to be transmitted between generations, which can constitute the basis of adaptative phenotypic plasticity [41, 42]. Moreover, epigenetic changes can be behind rapid adaptive changes observed in scenarios such as climate change, biological invasions and coevolutionary interactions. However, we need to disentangle the epigenetic variation from the genetic one and for that we need approaches that allow us to decrease genetic background (e.g., self-fertilization breeding) and introduce epigenetic changes (e.g., epidrugs, gene knockouts).

Our results hint at epimutations being a source of phenotypic variance that can be induced by chemicals that disrupt normal mechanisms of methylation control. In addition, this disruption may act on the germline, with phenotypic expression in the form of heightened phenotypic and epigenetic variance in the next generation. However, we have no proof that variation in methylation patterns is the only source of the variance in the phenotype found in F0 and F1 generations and we cannot formally exclude concomitant genetic variation. However, Flv1 could be used to induce epimutations in inbred self-fertilization lines and cross snails with divergent methylomes due to chemical treatment (e.g., hypomethylated versus hypermethylated snails) to create epigenetic recombinant inbred lines (epiRILs) as those created in *Arabidopsis thaliana* [44]. In this way, one can evaluate if, in the absence of genetic variation, epimutations and phenotypic variation induced in the exposed generations are transmitted across multiple generations and produce phenotypes having a selective advantage.

## Methods

### Ethics statement

*B. glabrata* adult snails of the albino Brazilian strain (*Bg*BRE) were used in this study. *P. acuta* juvenile individuals were raised in the Centre d’Ecologie Fonctionnelle et Evolutive CEFE UMR 5175 in Montpellier, France*. C. gigas* juveniles’ oysters were a generous gift of Bruno Petton from the Marine Mollusks Platform IFREMER in Bouin, France. *B. glabrata* mollusks were maintained at the IHPE laboratory facilities; they are kept in aquariums and fed with lettuce ad libitum. *C. gigas* and *P. acuta* mollusks were maintained during the 10 days of drug exposure in the quarantine room at the IHPE laboratory to avoid contact with the home breeding species (*B. glabrata* strains). The Direction Départementale de la Cohésion Sociale et de la Protection des Populations (DDSCPP) provided the permit N°C66-136-01 to IHPE for experiments on animals. Housing, breeding, and animal care were done following the national ethical requirements.

### Chemical stability measurement of the new DNMT inhibitors

The stability of the flavanone compounds Flv1, Flv2 and Flv-neg corresponding to compounds MLo1507 (3b), Flv880 (880) and MLo1607 (19) in [65] was measured by High-Performance Liquid Chromatography (HPLC) by the method described in [65]. HPLC analysis were done using an X-terra column (100 × 4.6 mm, 5 µm) with 1 mL/min flow and the following gradient: H_2_O acetonitrile 95:5 for 2 min then up to 0:100 in 10 min and maintained at 0:100 for 2 min with H_2_O and acetonitrile containing 0.1% of trifluoroacetic acid. First, flavanone compounds (Flv1, Flv2 and Flv-neg) were injected in solution at 100 µM in 100% DMSO to check its purity. Then, 50 µL of solution at 10 µM of tested compound was prepared by dilution in DNMT3A-c enzyme buffer (Hepes 20 mM pH 7.2, KCl 50 mM, EDTA 1 mM final concentration), in freshwater used in the aquariums of *B. glabrata* or in filtered sea water used in the aquariums of *C. gigas*. The percentages of remaining compound were determined with the area of the corresponding HPLC peak on the 250 nm chromatogram.

### *Bge* nuclear fraction extraction

Nuclear fractions were prepared by collecting *Bge* cells (the embryonic cell line of our model *B. glabrata*) by centrifugation, then cell pellet was lysed with a dounce homogenizer (7 mL) for 10 min at room temperature with cold 10 mM HEPES pH 7.7, 10 mM KCl, 0.1 mM EDTA, 1 mM DTT, and 0.4% IGEPAL CA-630 in the presence of protease inhibitors. The lysed cells were centrifuged at 15,000 × g for 3 min and the soluble fractions removed. The pellet was resuspended in 20 mM HEPES pH 7.7, 0.4 M NaCl, 10% glycerol, 1 mM DTT in the presence of protease inhibitors by vortexing for 2 h at 4 °C, followed by centrifugation at 15,000 × g (5 min, 4 °C) to provide the nuclear fractions (supernatant) and a membrane pellet. The nuclear fractions were quantified with the 2D Quant Kit (GE Healthcare Life Sciences, USA) and then stored at − 80 °C until use.

### DNMT inhibition methyltransferase activity on *Bge* nuclear extracts

Flv 1 inhibition activity was determined on *Bge* nuclear extracts with a fluorescence-based assay [13]. In brief, a double-strand DNA with a unique CpG site overlaying an endonuclease restriction site for methylation-sensitive enzyme was used. This oligonucleotide comprises a 6-carboxyfluorescein (6-FAM) at one end and biotin on the other end allowing immobilization into a 384-well plate (PerkinElmer) pre-coated with avidin. Flv1 and AdoMet as methyl donor were added followed by DNMT3A-c to start the methylation reaction, which was prolonged 1 h at 37 °C. After several washing, with PBS tween (0.05%) containing NaCl (0.5 M) and PBS tween (0.05%). Restriction step was performed with HpyCH4IV (New England, BioLabs) to hand on only the specific fluorescence signal. Fluorescence was quantified on a spectrofluorometer SAFAS FLX-Xenius. Methylation activities are defined as [(X_meth_-X_restri_)/(X_DNA_-X_restri_] × 100, where X_meth_, X_restri_ and X_DNA_ are, respectively, the fluorescence signals of the compound methylation, restriction, and DNA controls.

### DNA methyltransferase inhibitor (DNMTi) treatments in *B. glabrata*

Three types of DNMT inhibitors were tested in the snail *B. glabrata*, the cytidine analogue zebularine (Sigma, France, Cat. No. 3690–10-6) and custom-made inhibitors, previously selected for their inhibitory activity against *h*DNMT1 and *h*DNMT3-c [13], [65].

The custom-made compounds consist in the active flavanones: Flv1, Flv2, and Flv-neg corresponding to compounds MLo1507 (3b), DD880 (880) and MLo1607 (19) in [65]. Stock solutions at 10 mM were made in ultrapure Milli-Q water and aliquoted and stored at − 20 °C for all compounds.

For each condition, 100 adult snails of the *B. glabrata* Brazilian strain (*Bg* BRE) of approximately the same age (8 weeks) and size (5–7 mm) were randomly assigned to treatment and control groups, the treatments were done with the DNMTi at a final concentration of 10 µM in 1000 mL of well water in a plastic container, a single aquarium was made within each treatment. The *Bg* BRE strain is not an inbred strain, it can show concomitant genetic variability [12]. The water was replaced once with fresh drug-containing water at the same concentration, the replacement was performed after 3 days and 22 h. After 10 days of exposure, the drug was removed and replaced by drug-free water. Snails were then raised in the plastic tank for 70 days, during which different life history traits were measured. Mortality was measured at days 3, 4, 6, 8 and then each week. The egg-capsules laid by the snails of the generation F0 were separated each week to raise the F1 generation in another plastic container, the fecundity was reported as a single measure of number of juveniles and total number of eggs per treatment. At day 70, snails of the generation F0 and F1 were collected, the shell width, shell height and weight of each snail were recorded to compare morphometric trait variations between treatments. Finally, snails were stored wrapped in aluminum sheets individually at − 20 °C.

### Flv1 treatment in *C. gigas* and *P. acuta*

Thirty individuals of *P. acuta* and 30 of *C. gigas* of 5–7 mm of diameter were raised as the control groups. Thirty individuals of *P. acuta* and 30 of *C. gigas* of the same size were exposed to the Flv1 inhibitor at a concentration of 10 µM. The water was replaced once with fresh water for *P. acuta* and with filtered sea water for *C. gigas* both containing Flv1 inhibitor at the same concentration, the replacement was done after 3 days and 22 h. After 10 days of exposure, snails and oysters were collected and stored in aluminum sheets individually at − 20 °C.

### Genomic DNA extraction

Zirconia/Silica beads and the NucleoSpin® Tissue Kit (Macherey–Nagel, Düren, Germany), a method developed to extract DNA from the Pacific oyster [19], were used for DNA extraction from whole body without shell of *B. glabrata *(*n* = 300, 30 per treatment), *P. acuta* (*n* = 60) and *C. gigas* (*n* = 60). Briefly, for the lysis phase, 180 µL of lysis buffer, 25 µL of Proteinase K (20 mg/mL) and 100 µg of zirconia/silica beads (BioSpec, USA, Cat. No. 11079110z) were added to samples that were submerged in liquid nitrogen and then shaken in a Mixer Mill (Retsch MM400) at a frequency of 30 Hz for 12 min. Then, an incubation in water bath at 56 °C during 1 h 30 was done.

After lysis, the NucleoSpin® Tissue Kit protocol was applied according to the manufacturer instructions. Elution was performed into a final volume of 100 µL elution buffer. The samples were stored at − 20 °C. DNA concentrations of all samples were quantified using a Qubit® 2.0 fluorometer (Invitrogen) and a fluorescence-based Qubit™ dsDNA BR Assay Kit (Invitrogen, Q32853).

### Global DNA methylation detection

#### Dot blot

Detection and quantification of DNA methylation in genomic DNA were performed by dot blot assays using an antibody against 5mC. Before large screening, we optimized the dot blot method with DNA extracted from HeLa cells as a positive control and unmethylated PCR products as negative control. Different concentrations of HeLa cells were spotted to test the sensitivity and linearity of the method. After standardization of the method, genomic DNA of the control and treated mollusks (100 ng in 5 µL per replicate for equal loading) were denatured with 0.3 M NaOH at 42 °C for 10 min and spotted on nitrocellulose membranes (Hybond®). The membranes were blocked in 5% powdered milk diluted in 1× TBS containing 0.05% Tween 20 (TBST) for 1 h 30 at room temperature. Then, the membranes were incubated with a 1:500 dilution ratio of anti-5mC antibody (Abcam, #ab73938) and 5% powdered milk in TBST for 1 h 30, followed by 3 × 10 washes with TBST and elliptical agitation. Then incubation with a 1:500 dilution ratio of HRP-conjugated Goat anti-mouse IgG secondary antibody (ClinicSciences, #AS111772) was done.

The antibody mixture was then removed, and the membrane was washed with TBST under elliptical agitation during 3 × 10 min. Lecture of the signal was performed using the SuperSignal™ West Pico Chemiluminescent system (Thermo Fisher Scientific, USA) and the ChemiDoc MP Imaging System. Finally, the densitometry of the 5mC was analyzed with the software ImageLab5.1. Detailed protocol of this method is found in our preprint [56].

#### ELISA-based 5mC quantification

Methylated DNA Quantification Kit (Colorimetric) (Abcam, ab117128) was used to determine global 5mC level in isolated genomic samples of mollusk controls (*n* = 15 for *B. glabrata* and *n* = 10 for *P. acuta* and *C. gigas*) and Flv1 treated (*n* = 15 for *B. glabrata* and *n* = 10 for *P. acuta* and *C. gigas*) according to manufacturer instructions. To quantify the absolute amount of methylated DNA, a standard curve was generated plotting the OD values versus the amount of positive control at each concentration point.

### Statistical analyses

The data of mean spot densitometry provided by the software ImageLab5.1 was normalized by the DNA amount (ng) to obtain a relative measure of the 5mC level. Then, we calculated the 5mC% using the following equation:

$${\text{5mC }}\% = \left[ {{\text{sample densitometry}}/{\text{ng}}} \right]/\left[ {{\text{positive control densitometry}}/{\text{ng}}} \right] \, \times {\text{ Positive control 5mC }}\%$$

where the positive control densitometry corresponds to 6.9 ± 1.2 per ng of HeLa cells [20].

Rstudio was used for statistical analysis. Since the data displayed non-normal distribution demonstrated by the Shapiro–Wilk test (*p* < 0.01), Kruskal–Wallis test was used to compare means between groups, then Dunn’s multiple comparisons test was applied to test significance of differences in means between the control group and the different treatments. The survival curves were compared by a Mantel–Cox test and the fecundity was measured as the number of offspring and the number of eggs laid by the snails. A contingency table was elaborated with the number of offspring, the non-hatched eggs and the total of eggs laid, then a Fisher’s exact test was done to test for significant differences between the treatments. Differences of the morphometric traits between treatments were examined by One-way ANOVA when data were normally distributed (Shapiro–Wilk test was used to verify this assumption) and have a common variance (Levene’s test was used to verify equal variances), Welch’s ANOVA was applied when the assumption of equal variances was violated, and Kruskal–Wallis test was used when ANOVA assumptions were not met. Pairwise comparisons were done using Tukey multiple pairwise-comparisons, pairwise *t* test and Wilcoxon rank sum test with continuity correction, respectively.

### Library preparation and high throughput reduced representation bisulfite sequencing by epiGBS

We used an existing protocol called epiGBS [30, 82], a reduced representation bisulfite-sequencing method for cost-effective exploration of DNA methylation and genetic variation designed for multiplexed high-throughput sequencing to maximize sample size while losing *loci*. epiGBS sequencing was performed with the snails exposed to the DNMTi that showed the most significant changes in the global 5mC%. Eight samples per treatment were sequenced from control group, Flv1-treated, and from the progeny of control and the Flv1-treated group. 32 DNA isolated samples were quantified with Qubit fluorometer with the dsDNA HS Assay Kit (Invitrogen). The concentration was homogenized in all samples to 10 ng/µL in a total volume of 35 µL. epiGBS library preparation was applied as described in the step-by-step most recent protocol [30]. Paired-end sequencing (2 × 150 bp) using an Illumina NextSeq™550 instrument at the Bio-Environment NGS Platform at the University of Perpignan.

### Bioinformatics epiGBS pipeline

We used the epiGBS2 pipeline (https://github.com/nioo-knaw/epiGBS2) to remove PCR duplicates and demultiplex samples. We took the filtered and demultiplexed reads from epiGBS2 pipeline to use the pipeline described in [60]. Adapter removing was done using TrimGalore! V06.5 [50], 30 nucleotides were removed from 3ʹ and 5ʹ end. Single-end reads were aligned to *B. glabrata* genome v BglaB1 from https://www.vectorbase.org/organisms/biomphalaria-glabrata without scaffolds < 5 kb with BSMAP Mapper [86]. Then single mapped reads were merged and used as input in BSMAP Methylation Caller to get a tabular file with cytosine and thymine counts that was used as input to calculate coverage and Frequency of C and T for subsequent analysis.

After alignment, we filtered the CpG sites covered by 8 or more reads and pairwise comparisons and differential methylated analyses were done between control and treated samples in individuals of the same generation (F0 and F1) using MethylKit [2]. The parameters to calculate the Differentially Methylated Cytosines (DMCs) in MethylKit were *q* value < 0.05 and > 15% methylation difference as described in Johnson and Kelly [45] and other less strict parameters adapted to identification of DMCs with weak methylation effect and to small sample sizes, *q* value = 1 and 4% of methylation differences as described in [80]. The visualization of DMCs was done in Integrative Genomics Viewer (IGV). Reference transcriptome of *B. glabrata* was uploaded with bigwig files to see the location of DMCs. Genomic feature annotation was done by visualizing each differential methylated DMCs. Promoter was arbitrarily defined as the region 2 Kb upstream of transcription start site (TSS). WGBS raw data were analyzed with the same pipeline for direct comparison with epiGBs libraries. CpG sites with 8X coverage were used to created bigwig profiles for visualization in IGV (data available at: https://zenodo.org/record/4277533). CpG sites within gene bodies covered by epiGBS and WGBS were extracted by customized R scripts and used to construct histograms and quantiles distribution.

### Bisulfite conversion

300 ng of DNA from 8 control snails and 8 Flv1-treated snails were bisulfite converted as described previously [10, 28, 38]. 2 µg of tRNA from baker’s yeast (*S. cerevisiae*) was added to each sample, 3 M NaOH was added to a final concentration of 0.3 M, and DNA was denatured at 42 °C for 20 min. Then, 240 µL of freshly prepared bisulfite solution (5.41 g of sodium metabisulfite + 7 mL of distilled water + 0.5 mL of diluted Hydroquinone [0.022 g/10 mL] was added to the denatured DNA samples and incubated in the dark during 4 h at 55 °C. After that, 200 µL of distilled water was added to the samples, and the total volume was transferred to an Amicon column (UFC501024, Millipore, and centrifugation was done at 12,000* g* during 5 min. The column was washed 3 times with 350 µL of distilled water and centrifugation at 12,000* g* during 5 min was done each time. Following this, 350 µL of 0.1 M NaOH was added to the DNA in the Amicon column, centrifuged at 12,000* g* during 5 min; subsequently, 350 µL of distilled water was added and a centrifugation at 12,000* g* for 5 min was done. 50 µL of 10 mM TRIS/Cl pH 7.5–8.0 was added to the DNA in the Amicon column and it has been incubated at room temperature during 5 min. Finally, the DNA was collected by centrifugation at 1000* g* for 3 min. DNA was stocked at -80 °C.

### Nested PCR amplification of bisulfite-converted DNA

Primers were designed for PCR amplification in a CpG-rich region of the first intron of the BGLTMP010125 gene using MethPrimer [53]. The external primers (forward ATTGTGTTTTTATTTTGATGGTTATGATA and reverse CCCCAAAACTTACAAAAACCTTAC) were used to amplify a region spanning 861 bp in the BGLTMP010125 gene (Scaffold 4692: 13866–14343). The internal primers used in the nested PCR were the forward primer AGTTTTTTTTATTTTGTATGTAGAGT and the reverse primer ATCCTTTCAAAAAACAAATCATATATC; that amplify an amplicon of 565 bp. The initial PCR amplification was performed using 1 μL of the bisulfite-converted gDNA samples as templates with external primer set as follows: 94 °C for 2 min, 5 cycles of 94 °C for 1 min, 50 °C for 2 min and 72 °C for 3 min, followed by 30 cycles of 94 °C for 30 s, 50 °C for 2 min and 72 °C for 1:30 min and finally 72 °C for 10 min. The nested PCR was performed on a tenfold dilution of the first PCR product using the internal primer set in the same conditions as for the first PCR. PCR products were separated by electrophoresis through 2% agarose gels to check for the specific amplification of each target gene. PCR products were sequenced by Sanger sequencing (Genoscreen, Lille, France). Sequence chromatograms were analyses as previously described [43] to measure T-peaks heights for unmethylated cytosines converted to thymines, and C-peaks heights for methylated cytosines, providing an estimate for the degree of methylation.

### Dual DNA and RNA extraction and RT-qPCR

DNA and RNA were extracted from the same samples (n = 8 per treatment) with TRIzol reagent (Sigma Life Science) according to manufacturer’s instructions. DNA was subsequently bisulfite converted as described previously and RNA was reverse transcribed to first-strand cDNA using Maxima H Minus First-Strand cDNA Synthesis Kit with dsDNase to remove contaminating genomic DNA and following manufacturer’s instructions (Cat. Num. K1682, ThermoFisher, Scientific). Real-time RT-qPCR analyses were performed using the LightCycler 480 System (Roche) in a 10 µL final volume comprising 5 µL of No Rox SYBR Master Mix blue dTTP (Takyon), 1.75 µL of ultrapure Milli-Q water, and 1 µL of each primer at a concentration of 1 µM. The primers used for the RT-qPCR are shown in Table 5. Two housekeeping genes were used to normalize the results, the 28S ribosomal protein gene and the αTubulin protein gene, the primers efficiencies were previously evaluated by amplifying four different dilutions of each couple of primers at the RT reaction (1:1, 1:10; 1:100 and 1:1000), a standard curve was generated and the efficiency was calculated with the equation (Efficiency of the amplification = 10^^(1/−slope)^, as earlier described [46]. The cycling program was: denaturation step at 95 °C for 2 min, 40 cycles of amplification (denaturation at 95 °C for 10 s, annealing at 58 °C for 20 s, and elongation at 72 °C for 30 s), with a final elongation step at 72 °C for 5 min. For each reaction, the cycle threshold (Ct) was determined using the second derivative method of the LightCycler 480 Software release 1.5.0 (Roche). Reactions without RT served as negative control for each sample (in duplicate) to exclude amplification of DNA. None of these negative RT reactions amplified the target. All PCR experiments were performed in duplicates (technical replicates). The mean Ct value of each reaction was calculated and the 2^−ΔΔCT^ method was applied to calculate relative gene expression, the geometric mean of the Ct values of two housekeeping genes (28S and α-Tubulin) were used to normalize gene expression. Corrected melting curves were checked using the Tm-calling method of the LightCycler 480 Software release 1.5.0.

**Table 5:** *Biomphalaria glabrata* gene-specific primers used to amplify gene fragments used in the RT-qPCR

Gene	Primer Sequence	Amplicon length	Primer efficiency
28S ribosomal protein	F: GCTGGCACGACCGCTCCTTT	100 bp	2.01
	R: TTTGAACCTCGCGACCCGGC		
α-Tubulin	F: CGACATCTGCCGCCGTAACCT	112 bp	2.04
	R: GGCGCCATCAAACCTGAGGGA		
BGLTMP010125-RA:	F: TTGCTGTGACTGTCAGTGTC	95 bp	1.90
	R: TAGACTCAATGGACGGTGGAC		

## Supplementary Information

**Additional file 1.** **Figure S1.** Structure, molecular weight (Mw) and molecular. **Table S1.** Mapping efficiencies of paired-end reads, total number of CpG sites, CpG covered by at least 8 reads, number of CpG sites methylated, percentage of CG methylated and sequencing depth of each sample. Formula ofDNMT inhibitors. **Table S2.** Differential Methylated Cytosines (DMCs) of the F0 generation of snails treated with Flv1. **Table S3.** Differential Methylated Cytosines (DMCs) of the F1 generation, offspring of the snails treated with Flv1. **Table S4.** Differential Methylated Cytosines (DMCs) of the F0 generation of snails treated with Flv1 (*q* value = 1, difference cutoff = 4%). **Table S5.** Differential Methylated Cytosines (DMCs) of the F1 generation, offspring of the snails treated with Flv1 (*q* value = 1, difference cutoff = 4%). **Table S6.** DMCs common in F0 and F1 generation (*q* = .05, cutoff = 15%). **Table S7.** DMCs common in F0 and F1 generation (*q* = 1, cutoff = 4%).

## Data Availability

All Flv substrates are available on request. Raw data are available at NCBI SRA PRJNA771265.

## Abbreviations

6-FAM: 6-Carboxyfluorescein
THUMP: THioUridine synthases, RNA Methyltransferases and Pseudo-uridine synthases
AdoMet: *S*-Adenosyl-l-methionine
5mC: 5-Methylcytosine
DNMTs: DNA methyltransferases
DNMTi: DNA methyltransferase inhibitors
5-AzaC: 5-Azacytidine
BgBRE: *Biomphalaria glabrata* Strain Brazil BRE
Bge: *Biomphalaria glabrata* Embryonic
